# Association of co-existing vitamin B6 and B12 deficiency with polyneuropathy, organomegaly, endocrinopathy, M protein, and skin changes (POEMS) syndrome: a case report

**DOI:** 10.1186/s12883-025-04069-9

**Published:** 2025-02-07

**Authors:** Stephan Hu, Sharon Brown-Kunin, Paul Martin, Yujie Wang

**Affiliations:** 1 Department of Neurology, University of Washington, Seattle, USA; 2 Department of Family Medicine, University of Washington, Seattle, USA; 3 Division of Hematology and Oncology, Department of Medicine, University of Washington, Seattle, USA; 4 Department of Neurology, Johns Hopkins University, Baltimore, USA

**Keywords:** POEMS, Vitamin B6, Vitamin B12, Peripheral neuropathy

## Abstract

**Background:**

Both vitamin B6 deficiency and vitamin B12 deficiency can present with symptoms that appear like polyneuropathy, organomegaly, endocrinopathy, M protein, and skin changes (POEMS) syndrome, with painful peripheral neuropathy and sensorimotor dysfunction. There are rare reports of an association between vitamin B12 deficiency and POEMS syndrome, and even rarer reports of an association between vitamin B6 deficiency and POEMS syndrome. To our knowledge, this is the first described case with deficiencies in both vitamin B6 and vitamin B12 in association with POEMS syndrome.

**Case presentation:**

A man in his 40s presented with fatigue, imbalance, and painful numbness and tingling. Initial evaluation revealed low vitamin B12 level, and he received oral and IV supplementation for one month with an improvement in vitamin B12 levels, but without improvement in symptoms. Further evaluation revealed both a vitamin B6 deficiency and an IgA lambda monoclonal spike, prompting further investigation and an eventual diagnosis of POEMS syndrome. He underwent an autologous stem cell transplant and has had improvement in his symptoms.

**Conclusions:**

Patients with POEMS syndrome may have symptoms that are difficult to distinguish from deficiencies in vitamin B6 or vitamin B12. Management of POEMS should include screening of vitamin B6 and B12 to ensure other possible associated causes of symptoms are appropriately treated.

## Background

Monoclonal gammopathies are diseases in which plasma cells proliferate and cause an elevation of monoclonal protein. The association between monoclonal gammopathies and neuropathy has been described since at least the 1970s, but the exact pathophysiology is not well understood [1, 2]. Gammopathies known to have associations with peripheral neuropathy include monoclonal gammopathy of undetermined significance (MGUS), multiple myeloma, immunoglobulin light chain (AL) amyloidosis, and POEMS syndrome [1]. Of note, 3–4% of the population over age 50 has a monoclonal gammopathy, so routine screening of patients with neuropathy may reveal a monoclonal protein that is incidental and unrelated to the neuropathy [1]. 

POEMS syndrome is a rare paraneoplastic syndrome with a name that is descriptive but not all encompassing of its findings [3]. Criteria for diagnosis of POEMS syndrome requires both major mandatory criteria, one of three other major criteria, and one of six minor criteria. (Table 1) Differentiating POEMS from MGUS is critical as treatment directed at the clonal population generally would not be recommended in MGUS [3]. 

**Table 1:** Major and minor criteria for diagnosis of POEMS

Mandatory Major Criteria (Both required) 1. Polyneuropathy 2. Monoclonal plasma cell proliferative disorder	
Other major criteria (At least 1 required) 1. Castleman disease 2. Sclerotic bone lesions 3. Vascular endothelial growth factor elevation	
Minor criteria (At least 1 required) 1. Organomegaly (spleen, liver, lymph nodes) 2. Extravascular volume overload (pleural effusion, ascites, peripheral edema) 3. Endocrinopathy (adrenal, thyroid, pituitary, gonadal, parathyroid, pancreatic) 4. Skin changes (hyperpigmentation, hypertrichosis, hemangiomata, acrocyanosis) 5. Papilledema 6. Thrombocytosis/polycythemia	
**Other associations (None required)** 1. Clubbing, weight loss, hyperhidrosis, pulmonary hypertension, diarrhea, low vitamin B12	

Vitamin B12 deficiency is neither a major nor minor criteria of POEMS but is listed as a known association with POEMS syndrome. A review of the literature shows case reports of vitamin B12 deficiency in association with POEMS syndrome, but there is sparce information on why vitamin B12 deficiency may be associated with POEMS syndrome. However, there is a larger pool of data describing the association between plasma cell dyscrasias and vitamin B12 deficiency, and one study found that 13.6% of patients with plasma cell dyscrasias had vitamin B12 deficiency [4]. Given the similarities between POEMS syndrome and plasma cell dyscrasias such as multiple myeloma, it could be postulated that the mechanisms in POEMS syndrome causing vitamin B12 deficiency may be similar. These mechanisms include comorbid development of pernicious anemia, interference of the vitamin B12 absorptive process by the M protein, and more rapid consumption of the body’s vitamin B12 cells by malignant plasma cells [4, 5]. 

To our knowledge, vitamin B6 deficiency in association with POEMS syndrome is much rarer than vitamin B12 deficiency in association with POEMS and has only been described in two cases [6]. Etiology of vitamin B6 deficiency in association with POEMS is unclear, but given its role in multiple metabolic processes, could similarly be due to more rapid consumption by malignant plasma cells.

In this case, a patient presented with symptoms of peripheral neuropathy initially attributed to vitamin B12 deficiency. However, his symptoms did not improve with sufficient supplementation and improvement in his vitamin B12 level, and on further assessment, he was found to have vitamin B6 deficiency and a monoclonal gammopathy and was ultimately diagnosed with POEMS syndrome. To our knowledge, co-existing vitamin B6 and vitamin B12 deficiencies with POEMS syndrome have not yet been described.

## Case presentation

A man in his 40s presented with fatigue, imbalance, and numbness and tingling. He first developed painful bilateral toe tingling and numbness one year prior to presentation, with symptoms gradually ascending to his shins in a symmetric fashion. Additional history included a normal varied diet with no known occupational or toxic exposures and no drug or alcohol use. Past medical history included obstructive sleep apnea. There was no history of gastric surgery, bowel disease, restricted diet or malnutrition, or chronic alcohol dependence. Review of systems revealed one and a half years of erectile dysfunction and six months of bilateral hearing loss, as well as sleep disturbance and mild impairment in short term memory. Family history included a mother with a history of myositis. On examination, he had fully intact cranial nerves and motor strength in the upper and lower extremities. Deep tendon reflexes (DTRs) were globally absent, except for 1 + at the right biceps tendon. He had absent proprioception and vibration distally in his legs, and hypersensitivity to pinprick in a stocking-distribution up the bilateral shins. He was able to walk independently, but his gait showed bilateral foot drop with steppage gait in addition to proprioceptive deficits leading to a cautious and imbalanced gait.

His symptoms were suspected to be most consistent with peripheral neuropathy. In the initial work-up, laboratory studies included normal complete blood count, complete metabolic panel, erythrocyte sedimentation rate, c-reactive protein, and creatinine kinase. Antinuclear antibodies were negative. Serum folate was 8.7 ng /mL (reference range > 5.8 ng/mL). Vitamin B12 was 72 pg/mL (reference range 180–914 pg/mL). The patient was diagnosed with vitamin B12 deficiency and started supplementation with weekly 1000 mcg vitamin B12 intramuscular injections and oral 1000 mcg vitamin B12 pills. Methylmalonic acid and homocysteine were not assessed prior to supplementation. Intrinsic factor and parietal cell antibodies were negative. Esophagogastroduodenoscopy showed esophagitis and colonoscopy showed normal appearance of the mucosa. Biopsies of the duodenum and stomach did not show active inflammation. Reassessment of his vitamin B12 level after supplementation for one month was within normal range at 423 pg/mL. Despite supplementation and maintaining adequate level of vitamin B12, he continued to have progression of his symptoms in the ensuing 6 months.

Further evaluation included normal hemoglobin A1c, normal TSH, negative HIV and syphilis serologies, normal vitamin E, and a low vitamin B6 level of 2 mcg/L (reference range 5–50 mcg/L). Supplementation for vitamin B6 was started at 100 mg daily. Serum monoclonal protein panel and immunofixation showed an IgA lambda monoclonal spike at 0.1 g/dL, serum free light chain panel demonstrated a kappa to lambda ratio within normal limits at 0.68, though lambda free light chains were elevated at 5.28. Electromyography and nerve conduction study (EMG/NCS) approximately one year into his symptoms showed findings consistent with a severe axonal length-dependent sensorimotor neuropathy, including absent bilateral fibular motor responses at the extensor digitorum brevis, absent bilateral tibial motor responses, reduced amplitude of the bilateral fibular compound muscle action potentials at the tibialis anterior, absent bilateral sural sensory responses, fibrillation potentials and positive sharp waves in the left tibialis anterior and gastrocnemius, and increased incidence of high amplitude motor unit action potentials and reduced motor unit action potential recruitment in the left tibialis anterior and gastrocnemius.

Given the progressive sensory symptoms in his legs, the patient underwent MRI of his lumbar spine, which showed no nerve root abnormalities, and no canal or neuroforaminal stenosis. However, there were multiple indeterminate foci of T1 hypointensities throughout the bony spine and pelvis. CT of the pelvis and lumbar spine were obtained, which showed numerous sclerotic lesions in the lower lumbar, pelvic, and bilateral femoral bones. Nuclear medicine bone scan showed no osseus metastatic disease.

**Fig. 1:**
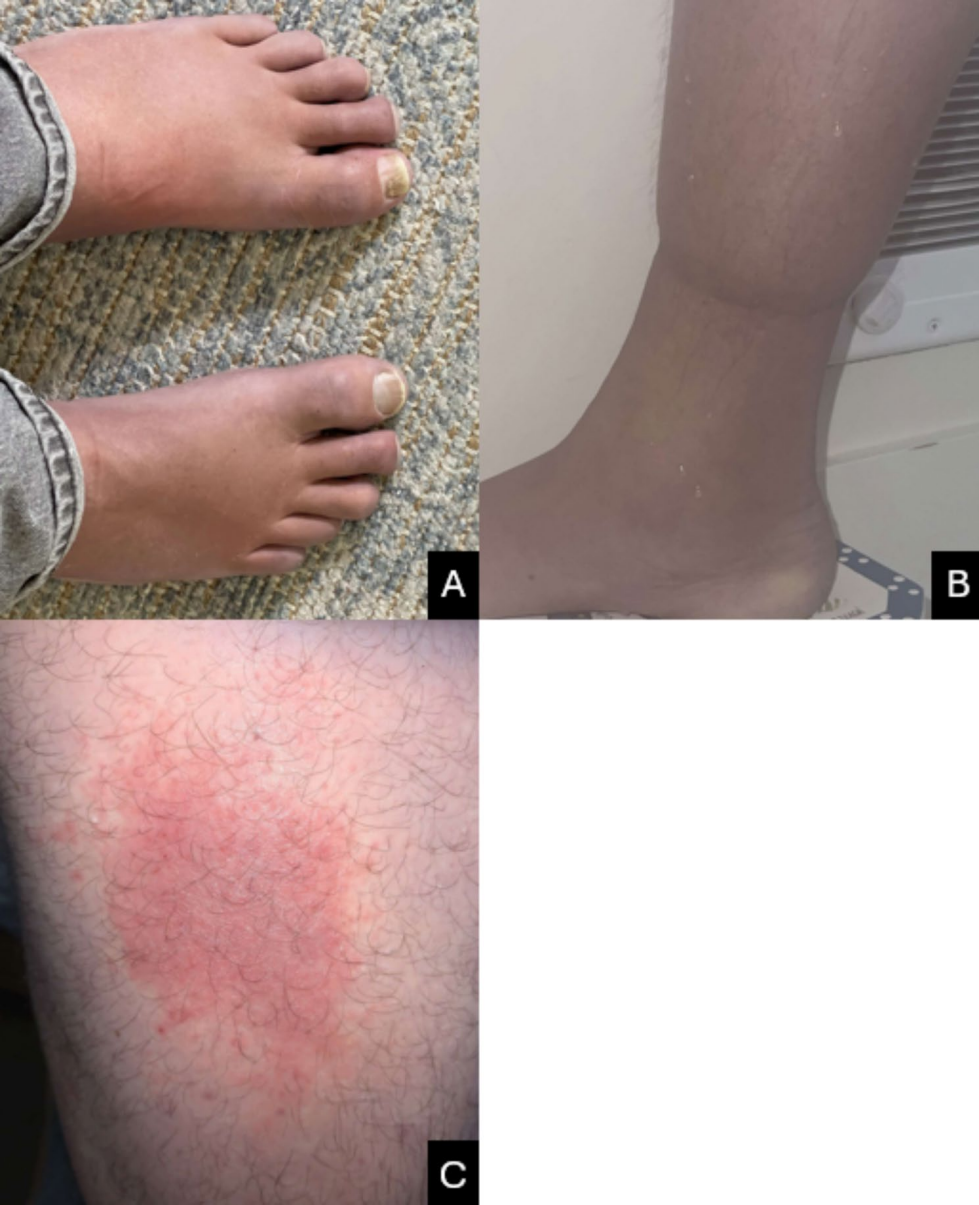
Examination findings. **A:** Acrocyanosis. **B:** Lower extremity pitting edema. **C:** Rash

Further evaluation of the patient revealed lower extremity edema and a history of skin changes (Fig. 1). There was no evidence of dermatitis, cheilosis, stomatitis or glossitis on exam. MRI enterography showed splenomegaly. Given the presence of both mandatory major criteria (polyneuropathy and monoclonal plasma cell proliferative disorder), at least one other major criteria (sclerotic bone lesions), and at least one minor criteria (skin changes, extravascular volume overload, endocrinopathy), as well as other associations (vitamin B12 deficiency), the patient was diagnosed with POEMS syndrome. Further evaluation revealed elevated vascular endothelial growth factor (VEGF) (121 pg/mL, reference range 9–86 pg/mL), low total testosterone (243 ng/dL, reference 264–916 ng/dL), and evidence of adrenal insufficiency on cortisol stimulation test. He also underwent fluorodeoxyglucose F-18 positron emission tomography/computerized scan metabolically active osseus lesions, as well as innumerable dense sclerotic lesions throughout the axial and appendicular skeleton without increased FDG uptake and diffuse anasarca. Bone marrow biopsy showed a hypercellular bone marrow with flow cytometry showing an abnormal plasma cell population (15–20% plasma cells by immunohistochemistry), consistent with POEMS syndrome. He underwent autologous stem cell transplantation. His lower extremity edema and skin lesions improved post-transplantation. Monoclonal protein panel with immunofixation continues to show IgA monoclonal spike. VEGF normalized from 121 pg/mL to 31 pg/mL. Vitamin B12 improved from 423 pg/mL to 822 pg/mL post-transplantation. Vitamin B6 improved from 11 mcg/L to 79 mcg/L, and supplementation was stopped given the supratherapeutic level (reference 5–50 mcg/L), he has continued to maintain a normal vitamin B6 level without supplementation. Post-transplant bone marrow biopsy showed minimal clonal plasma cells (0.05%, decreased from 1.5% of the total white cells pre-transplant). He continues to work on physical rehabilitation and pain control and has had gradual improvements in his neurologic symptoms in the 14 months post-transplant follow up period.

## Discussion and conclusions

In this case, we describe a patient who presented with symptoms of peripheral neuropathy and was ultimately diagnosed with and treated for POEMS syndrome. He was found to have both comorbid vitamin B12 deficiency and vitamin B6 deficiency, which are not frequently described as both being associated with POEMS syndrome.

The association between peripheral neuropathy and POEMS syndrome is well described, as is the association between peripheral neuropathy and vitamin B12 deficiency. Peripheral neuropathy has also been described in association with vitamin B6 but is more commonly thought to cause neuropathy in excess, not in deficiency [7]. However, there is some literature suggesting that vitamin B6 deficiency may be correlated with neuropathy when comorbid with other conditions, such as diabetes or concurrent vitamin B12 deficiency [8, 9]. 

Interestingly, there is also a report detailing rapid recovery of polyneuropathies in two patients with POEMS syndrome after repletion of low vitamin B6 [6]. This is a stark contrast to the typically slow improvement of polyneuropathy in POEMS, and could imply that at least part of the underlying mechanism causing neuropathy in POEMS may be related to vitamin B6 deficiency, and that an evaluation of vitamin levels in POEMS may be currently underappreciated. In contrast, a larger case series of patients who underwent autologous stem cell transplant also showed improvement, and in this series, vitamin B6 levels were not even measured [10]. Taken together, this could suggest that the deficiency in vitamin B6 seen in these cases is a downstream effect of the clonal plasma cell population and may be contributory to the development of neuropathic symptoms.

In this case, our patient’s EMG/NCS showed a severe axonal length-dependent sensorimotor neuropathy, which can be atypical of POEMS syndrome. Findings more suggestive of demyelination such as decreased motor conduction velocity, prolonged distal latency, or prolonged F-wave latencies were not seen. Possible explanations for the axonal features include the severity of his peripheral neuropathy with evidence of absent motor responses, which can conceal superimposed demyelinating features, as well as the co-existing vitamin B6 and vitamin B12 deficiencies. POEMS syndrome is thought to be a progressive demyelinating sensorimotor polyneuropathy with superimposed or secondary axonal loss [2]. Given these features, it can be mistaken for chronic inflammatory demyelinating polyneuropathy (CIDP), and in fact, up to 60% of POEMS patients may be initially misdiagnosed with CIDP and receive CIDP-directed treatment [11]. Whereas CIDP typically presents with demyelinating features, EMG/NCS of POEMS syndrome typically shows both axonal loss and demyelinating features, with more marked features of axonal loss [11]. This may be further muddied by the existence of vitamin B6 and vitamin B12 deficiencies, which may also mimic some of the EMG/NCS findings seen in POEMS syndrome.

Given all this information, it may be of use to screen for vitamin deficiencies such as vitamin B12 and vitamin B6 deficiencies in patients diagnosed with POEMS syndrome, as supplementation may assist in eventual improvement of neuropathic symptoms. In addition, in individuals whose neuropathies are attributed to vitamin deficiencies but do not improve/worsen despite sufficient supplementation, alternative causes should be considered, including POEMS syndrome.

## Data Availability

No datasets were generated or analysed during the current study.

## Abbreviations

POEMS: Polyneuropathy, Organomegaly, Endocrinopathy, M protein, Skin changes
